# Pyrochlore Compounds From Atomistic Simulations

**DOI:** 10.3389/fchem.2021.733321

**Published:** 2021-11-03

**Authors:** Timothy Connor, Oskar Cheong, Thomas Bornhake, Alison C. Shad, Rebekka Tesch, Mengli Sun, Zhengda He, Andrey Bukayemsky, Victor L. Vinograd, Sarah C. Finkeldei, Piotr M. Kowalski

**Affiliations:** ^1^ Department of Chemistry, Chemical and Biomolecular Engineering, and Materials Science and Engineering, University of California, Irvine, Irvine, CA, United States; ^2^ Forschungszentrum Jülich GmbH, Institute of Energy and Climate Research - IEK-13, Theory and Computation of Energy Materials, Jülich, Germany; ^3^ Jülich Aachen Research Alliance, JARA Energy & Center for Simulation and Data Science (CSD), Jülich, Germany; ^4^ Chair of Theory and Computation of Energy Materials, Faculty of Georesources and Materials Engineering, RWTH Aachen University, Aachen, Germany; ^5^ Walter Scott Jr. College of Engineering, Colorado State University, Fort Collins, CO, United States; ^6^ School of Nuclear Science and Technology, Lanzhou University, Lanzhou, China; ^7^ Forschungszentrum Jülich GmbH, Institute of Energy and Climate Research - IEK-6, Nuclear Waste Management and Reactor Safety, Jülich, Germany

**Keywords:** pyrochlores, atomistic simulations, energy storage materials, ceramics, solid solutions, solid state electrolyte, radiation damage

## Abstract

Pyrochlore compounds (*A*_2_ *B*_2_O_7_) have a large applicability in various branches of science and technology. These materials are considered for use as effective ionic conductors for solid state batteries or as matrices for immobilization of actinide elements, amongst many other applications. In this contribution we discuss the simulation-based effort made in the Institute of Energy and Climate Research at Forschungszentrum Jülich and partner institutions regarding reliable computation of properties of pyrochlore and defect fluorite compounds. In the scope of this contribution, we focus on the investigation of dopant incorporation, defect formation and anion migration, as well as understanding of order-disorder transitions in these compounds. We present new, accurate simulated data on incorporation of U, Np, Pu, Am and Cm actinide elements into pyrochlores, activation energies for oxygen migration and radiation damage-induced structural changes in these materials. All the discussed simulation results are combined with available experimental data to provide a reliable description of properties of investigated materials. We demonstrate that a synergy of computed and experimental data leads to a superior characterization of pyrochlores, which could not be easily achieved by either of these methods when applied separately.

## 1 Introduction

Pyrochlore-type ceramics are functional materials with a wide range of applications, including ionic conductors (Wuensch, 2000; Diazguillen et al., 2008; Kumar et al., 2008; Mandal et al., 2008; Shlyakhtina and Shcherbakova, 2012; Anithakumari et al., 2016), coatings (Feng et al., 2013), luminescent materials (Park et al., 2001; Öztürk et al., 2020) or matrices for immobilization of nuclear waste (Ringwood et al., 1979; Wang et al., 1999; Ewing et al., 2004; Bosbach et al., 2020). Significant research effort has been devoted to the complex characterization of these materials in order to understand their physical and chemical properties that make them multi-functional compounds (e.g., Sickafus et al., 2000; Ewing et al., 2004; Ushakov et al., 2007; Li et al., 2015; Uberuaga et al., 2015; Chung et al., 2018a; Maram et al., 2018; Drey et al., 2020). On many occasions, atomistic simulations have supported the interpretation of experimentally seen phenomena and a joint experimental and simulation-based research effort has led to an enhanced understanding of the experimental data (e.g., Finkeldei et al. (2017); Kowalski (2020)). There is a vast amount of past research on pyrochlore-type ceramics that involve various experimental, theoretical and computer-aided research techniques. Here we focus on discussing and reporting results of atomistic simulation efforts with which we have contributed to enhanced, although still far from being complete, understanding of various aspects of these materials.

**FIGURE 1:**
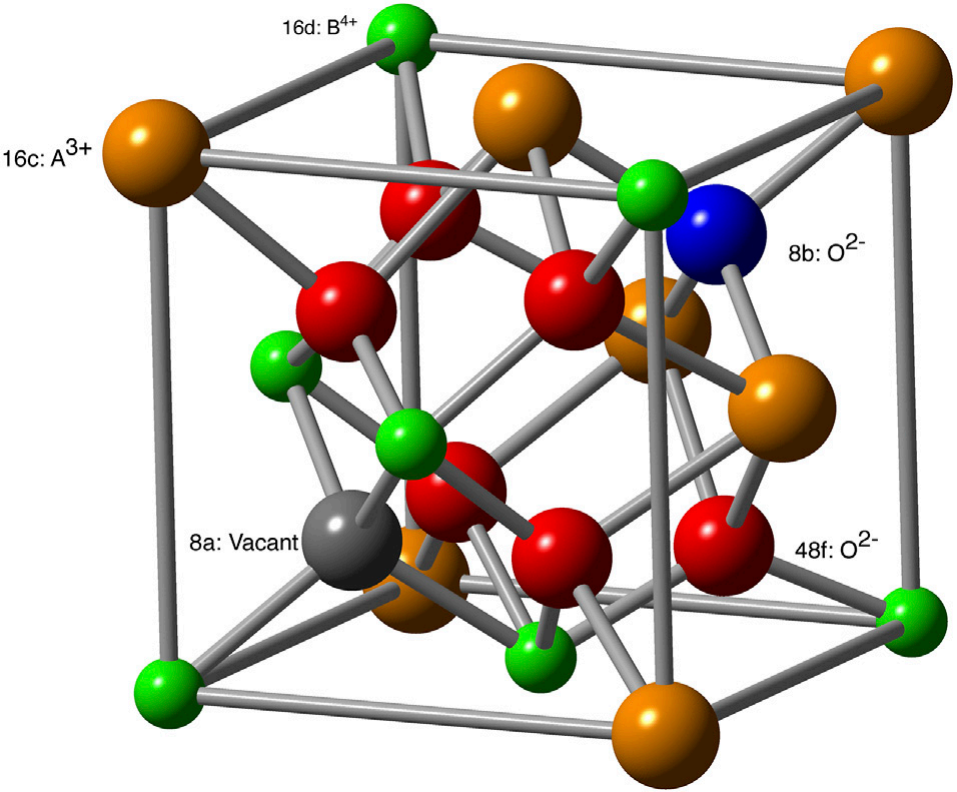
Structure of *A*_2_ *B*_2_O_7_ pyrochlore. Different crystallographic positions are labeled. For anion sublattice these are also indicated by different colors: 48*f* (red), 8*a* (gray, vacant site in stoichiometric pyrochlore) and 8*b* (blue).

Pyrochlores are crystalline solids (*A*_2_ *B*_2_O_7_) with the Fd-3m space group (227) and represent a superstructure of the fluorite structure (Ewing et al., 2004). In these compounds the *A* cation is typically a trivalent rare earth metal, and the *B* cation is typically a tetravalent transition metal element. The structure of one eighth of a stoichiometric pyrochlore unit cell is given in Figure 1. The *A*-site is 8-fold coordinated on the 16*c* Wyckoff position, and the *B*-site is 6-fold coordinated at the 16*d* position. The oxygen anions occupy the 48*f* and 8*b* positions. *A*-site cations are coordinated to six 48*f* and two 8*b* oxygens, with the *A*-48*f* bond lengths slightly longer than the *A* − 8*b* bonds. The *B*-site cations are coordinated to six 48*f* oxygens, and are adjacent to the vacant 8*a* sites.

In the last two decades, atomistic modeling became a widely used research technique in various research fields, including pyrochlores (Sickafus et al., 2000; Chroneos et al., 2013; Jahn and Kowalski, 2014; Li et al., 2015; Uberuaga et al., 2015; Wu et al., 2019). We used it intensively over the past decade for computation of various physical and chemical properties of pyrochlore-type ceramics. In particular, the computer-aided simulations have been applied to understand defect formation (Li et al., 2015; Kowalski et al., 2016; Li et al., 2016; Li and Kowalski, 2018), disordering behavior (Finkeldei et al., 2017; Kowalski, 2020), ionic conductivity (Li and Kowalski, 2018; Bukaemskiy et al., 2021) and structural incorporation of actinide elements (Finkeldei et al., 2020) in this class of materials. Besides our efforts, many other computational studies have substantially contributed to understanding different properties of these materials. In one of the first application of atomistic modeling to pyrochlores, van Dijk et al. (1985) found two distinct vacancy defect configurations, which is helpful in understanding the ability of these materials to conduct oxygen ions (e.g., Pirzada et al. (2001); Li and Kowalski (2018)). In a series of papers, Sickafus et al., 2000; Sickafus et al., 2007; Minervini et al., 2002; Minervini et al., 2000 applied force-field-based simulations to understand defect formation and disordering tendencies in pyrochlores. This effort was later followed by related *ab initio* simulations (e.g., Panero et al., 2004; Jiang et al., 2009; Vanpoucke et al., 2011; Li et al., 2015). Structural transformations under irradiation have been investigated by classical and *ab initio* molecular dynamics methods (Chartier et al., 2003; Chartier et al., 2005; Chartier et al., 2009; Crocombette and Chartier, 2007; Xiao et al., 2015). These are just a few examples of successful applications of atomistic modeling techniques to the research on pyrochlore-type ceramics.

The tendency of pyrochlores to disorder, and the ordering of the state they eventually reach, is an important, widely investigated aspect that impacts, for instance, radiation damage resistance (where the ability to form a disordered phase can prevent total amorphization) or ionic conductivity. Until recently, many studies assumed that pyrochlore disorders to an ideal defect fluorite, a crystalline solid in which cations and oxygen/oxygen vacancy distributions are completely random (e.g., Jiang et al. (2009); Li et al. (2015)). This concept has been successfully applied by Jiang et al. (2009) to derive the disordering transition temperature for a series of pyrochlore hafnates and zirconates using the concept of Special Quasi-random Structure (SQS) (Zunger et al., 1990) to model the disordered pyrochlore as perfectly disordered defect fluorite phase. The Density Functional Theory (DFT)-based simulations give a reasonably good agreement with measured values, although with an offset of ∼ 300 K. This has been corrected by application of the DFT + *U* method, with the self-consistently derived Hubbard *U* parameter values, that better accounts for the electronic correlations (Li et al., 2015). It has been demonstrated by calorimetric measurements (Ushakov et al., 2007; Saradhi et al., 2012; Finkeldei et al., 2017) as well as by neutron total scattering experiments (Shamblin et al., 2016) that disordered pyrochlore compounds retain a significant amount of local order, called short range ordering (SRO). The later studies found that such SRO can be well described with long-range ordered weberite model. This has been supported by the atomistic simulations of Kowalski (2020), who has shown that fully disordered compounds would be thermodynamically unstable against mixture of relevant oxides, and that the weberite-type structural representation of disordered pyrochlore results in computed formation enthalpies that match the existing measurements reasonably well.

Several pyrochlore-type compounds are considered as fast ionic conductors (Mandal et al., 2008; Diazguillen et al., 2008; Anithakumari et al., 2016; Moon and Tuller, 1988). In general, zirconia-based compounds are known to show high ionic conductivity. Among these, yttria-stabilized zirconia is considered as one of the fastest ionic conductors (Ahamer et al., 2017; Kowalski et al., 2021). In pyrochlore-type compounds, the ionic conduction happens through hopping between 48*f* sites. In an ideal pyrochlore these sites are fully occupied (Figure 1). In order to allow conduction, vacancies must be formed on the 48*f* sites (van Dijk et al., 1985; Pirzada et al., 2001; Li and Kowalski, 2018). Typically, this occurs via transfer of an oxygen atom to the vacant 8*a*-site and formation of an anion Frenkel pair defect. For pyrochlores containing heavier lanthanides, the oxygen diffusion path involves formation of a split vacancy state (van Dijk et al., 1985; Li and Kowalski, 2018). Li and Kowalski (2018) have shown that this state stabilizes the vacancy and leads to a significant increase of the activation barrier for diffusion and decrease of the ionic conductivity. This is consistent with the experimental measurements of ionic conductivity in zirconate-pyrochlores (Yamamura, 2003). Bukaemskiy et al. (2021) constructed a model for oxygen diffusion in zirconia doped with trivalent elements, in which the oxygen conductivity was assumed proportional to the probability of a vacancy jump between clusters in a certain typical configuration occurring more frequently due to SRO. Such a model has been successfully applied together with atomistic simulations to derive the ionic conductivity in yttria-stabilized zirconia materials (Kowalski et al., 2021). Here, with simulations of energies of defects formation on anion sublattice and activation barriers for oxygen diffusion we will discuss the mechanisms of ionic conductivity in a series of zirconate pyrochlores.

Another important aspect of pyrochlores is their ability to effectively immobilize radionuclide elements such as Pu and minor actinides (Np, Am, Cm) (Ewing et al., 2004; Bosbach et al., 2020). A matrix of pyrochlore-type is thus considered as a durable nuclear waste form to increase safety of radioactive materials permanently disposed in a deep geological repository. An important aspect of such a strategy is non-proliferation, by immobilizing large stockpiles of weapons grade Pu in a form of durable material (Ewing et al., 2004). Understanding the mechanism of structural incorporation of actinides into pyrochlore ceramics is of utmost importance in assessing the performance of pyrochlores as nuclear waste forms. In that respect, Nästren et al. (2009) measured the lattice parameter and Extended X-ray Absorption Fine Structure (EXAFS) spectra of various Nd_2_Zr_2_O_7_ pyrochlores doped with different actinide elements (Th, U, Np, Pu, Am) in various oxidation states, and concluded that these actinides were incorporated on the *A* (Nd) site. With the aid of atomistic simulations Ji (2018) demonstrated that the lattice parameter change upon doping with actinide elements resembles the experimental data only when assuming doping on *A* cation site (Nd), regardless of the actinide valence state. Here we applied more accurate computational methodology to confirm this hypothesis. More recently, applying a combination of EXAFS and atomistic simulations Finkeldei et al. (2020) demonstrated that Pu incorporates in the same way as Pu(IV) species, although EXAFS measurements could possibly also indicate distribution of Pu between Nd and Zr cation sites. Another aspect of using pyrochlores as nuclear waste form is their resistance to radiation damage (e.g., Gd_2_Zr_2_O_7_, Ewing et al. (2004)). This behavior is associated with a thermodynamic easiness of an order/disorder transformation and transformation to defect fluorite phase (disordered pyrochlore), aspects which, as aforementioned, have been in focus of series of atomistic modeling studies as well as irradiation experiments (e.g., Lian et al. (2006b); Chung et al. (2018a); Crocombette et al. (2006); Chartier et al. (2009)).

In this contribution we provide an overview of recent atomistic modeling activities on the pyrochlore-type ceramics, focusing on information that has been delivered by activities at Forschungszentrum Jülich, and that allowed on many occasions for better characterization of these materials, including structural data, thermodynamic stability and disordering tendencies. As a novelty we present computational results on: structural parameters of pyrochlores doped with actinides, activation barriers for ionic conduction in this class of materials and behavior of pyrochlores under irradiation. We highlight a cross-linking, interdisciplinary character of the research on pyrochlores, from which the general science community could highly benefit.

## 2 Computational Approach

The here reported *ab initio* *^1^* calculations were performed with the DFT-based Quantum-ESPRESSO package (Giannozzi et al., 2009). It consists of an integrated suite of computer codes capable of nanoscale materials modeling, including electronic structure calculations. Most importantly for the studies on pyrochlores, it allows for the state-of-the-art application of DFT + *U* approach to improve description of correlation effects among *f* electrons. We applied the PBE and PBEsol exchange-correlation functionals (Perdew et al., 1996; Perdew et al., 2008). The energies were computed with the PBE functional, while the PBEsol functional has been applied in the structural investigation. This is because by correctly reproducing the slowly varying electron density limit, when compared with the PBE approximation, PBEsol results in much better description of structural parameters of solids (Perdew et al., 2008), although at the cost of a lower accuracy of the computed energies. Application of PBEsol exchange-correlation functional is especially important for consideration of structural incorporation of elements into the pyrochlore phases. The core electrons of atoms have been represented by the ultrasoft pseudopotentials (Vanderbilt, 1990), with the relevant plane-wave energy cutoff of 50 Ryd. Following our broad experience on computation of lanthanide orthophosphates and zirconates, and uranium-oxides (e.g., Beridze and Kowalski, 2014; Blanca-Romero et al., 2014; Li et al., 2015; Beridze et al., 2016; Kowalski et al., 2021) here we applied a parameter free DFT + *U* approach. In this method, the Hubbard *U* parameter values are computed from first principles using the linear response method of Cococcioni and de Gironcoli (2005). This computational setup has been extensively tested by us in several studies and, among others, proved to give very accurate results for *Ln*-O bond lengths (Blanca-Romero et al., 2014; Beridze et al., 2016). For the simulations of oxygen migration and defect formation energies we applied the *“f in the core”* approach, in which *f* electrons are not computed explicitly, but their presence is simulated by pseudopotentials. These simulations are more stable and give results that are comparable to these obtained with the outlined DFT + *U* approach for materials properties that do not directly involve *f* elements (see discussions in Blanca-Romero et al. (2014) and Beridze et al. (2016)).

The activation barriers were computed using the Nudged Elastic Band (NEB) method as implemented in Quantum-ESPRESSO package. The climbing image method and a set of five images were applied to compute the transition state and relevant activation barriers.

The simulations of radiation damage were performed with the aid of force-field-based molecular dynamics simulations using the LAMMPS code (Plimpton, 1995), using the random cation displacement procedure applied by us recently to simulations of irradiated borosilicate glasses (Sun et al., 2021). Similar procedure has been applied in previous atomistic simulation studies of pyrochlores (Chartier et al., 2005; Ji, 2018). Here, we simulated systems containing 2376 atoms (216 formula units) and the interatomic interactions were described by the simple Buckingham-type interaction potential of Minervini et al. (2000) and Stanek et al. (2002). The damage accumulation molecular dynamics simulations were performed in an iterative loop, with intervals between single cation defect formation of 2 ps. Within this short time, the NPT (constant pressure-temperature) simulations were performed assuming ambient condition. These were followed by a displacement of a randomly selected cation in random direction and distance, so that the displacement distance was at least 6 Å (to assure formation of a permanent defect).

## 3 Results and Discussion

### 3.1 Structural Data

#### 3.1.1 Stoichiometric Pyrochlores

Correct prediction of structural parameters by a simulation method is a key factor for atomistic modeling-based characterization of materials. The force fields used in classical molecular dynamics simulations are often parameterized to reproduce structural parameters of investigated materials, and this has been also the case in the research on pyrochlores (e.g. Minervini et al., 2000). In similar way, the *ab initio* and, in principle, DFT simulations, are also expected to provide a good description of the structures of solids. However, different applied approximations (e.g., exchange-correlation functionals) result in different quality of the computed structural data (Perdew et al., 2008; Blanca-Romero et al., 2014). In a series of papers on orthophosphates we demonstrated that a very good match to the structural parameters, especially the *Ln*-O bond lengths, can be obtained with the PBEsol exchange-correlation functional (Perdew et al., 2008) and the DFT + *U* method with the Hubbard *U* parameter derived from first principles (Blanca-Romero et al., 2014; Beridze et al., 2016; Kowalski et al., 2021). Finkeldei et al. (2017) have shown that PBEsol functional can predict accurate value of the lattice parameter of the Nd_2_Zr_2_O_7_ pyrochlore. We note that those calculations were performed with the *f in the core* approach (see Section 2). With the PBEsol exchange-correlation functional and the outlined parameter-free DFT + *U* method Blanca-Romero et al. (2014) got excellent results for the *Ln*−O bond-lengths in and volumes of *Ln*PO_4_ compounds. Here we test if such a method could predict good lattice parameters and *Ln*−O bond-lengths for series of zirconate-pyrochlores. In Table 1 we provide a set of the Hubbard *U* parameters computed for different lanthanide cations. The values are consistent with the previous simulations of Li et al. (2015) for pyrochlores and the aforementioned results of Blanca-Romero et al. (2014) for lanthanide phosphates. There is a clear trend that reflects strength of the electronic correlations for different *Ln* cations. The Hubbard *U* parameter increases along lanthanide series, reaching maximum for Eu and then becomes smaller for Gd. This is an effect of completely filled half *f*-shell of Gd^3+^.

**TABLE 1:** The computed Hubbard *U* parameter values and *Ln* − O bond lengths *d* (computed with the DFT *”f in the core”* and DFT + *U* methods), reported as differences from the measured bond-lengths (last column, data from Harvey et al. (2005); Koohpayeh et al. (2014); Vaisakhan Thampi et al. (2017); Hagiwara et al. (2013); Mandal et al. (2007); Nästren et al. (2009); Sasaki et al. (2004)), for *Ln* cations in *Ln*_2_Zr_2_O_7_ pyrochlores. The lengths of short/long bonds are reported, with the average error (AE) provided in the last row.

Compound	*U* Parameter (eV)	$\Delta d_{\ln-O}^{DFT+U}$ ( $\overset{{}^{\circ}}{A}$ )	$\Delta d_{\ln-O}^{DFT}$ ( $\overset{{}^{\circ}}{A}$ )	$d_{ln-O}^{\exp}$ ( $\overset{{}^{\circ}}{A}$ )
La	2.8	−0.006/−0.002	0.000/−0.002	2.339/2.642
Ce	3.7	−0.003/−0.003	−0.002/−0.003	2.327/2.614
Pr	4.3	−0.006/−0.009	−0.005/−0.010	2.317/2.596
Nd	5.0	−0.006/−0.005	−0.009/−0.016	2.311/2.583
Pm	5.1			
Sm	6.3	0.008/−0.085	−0.005/−0.099	2.290/2.631
Eu	7.6	0.016/0.014	−0.007/−0.007	2.285/2.524
Gd	3.9	−0.001/0.040	−0.011/0.018	2.282/2.485
AE		0.003/0.028	0.006/0.022	

**FIGURE 2:**
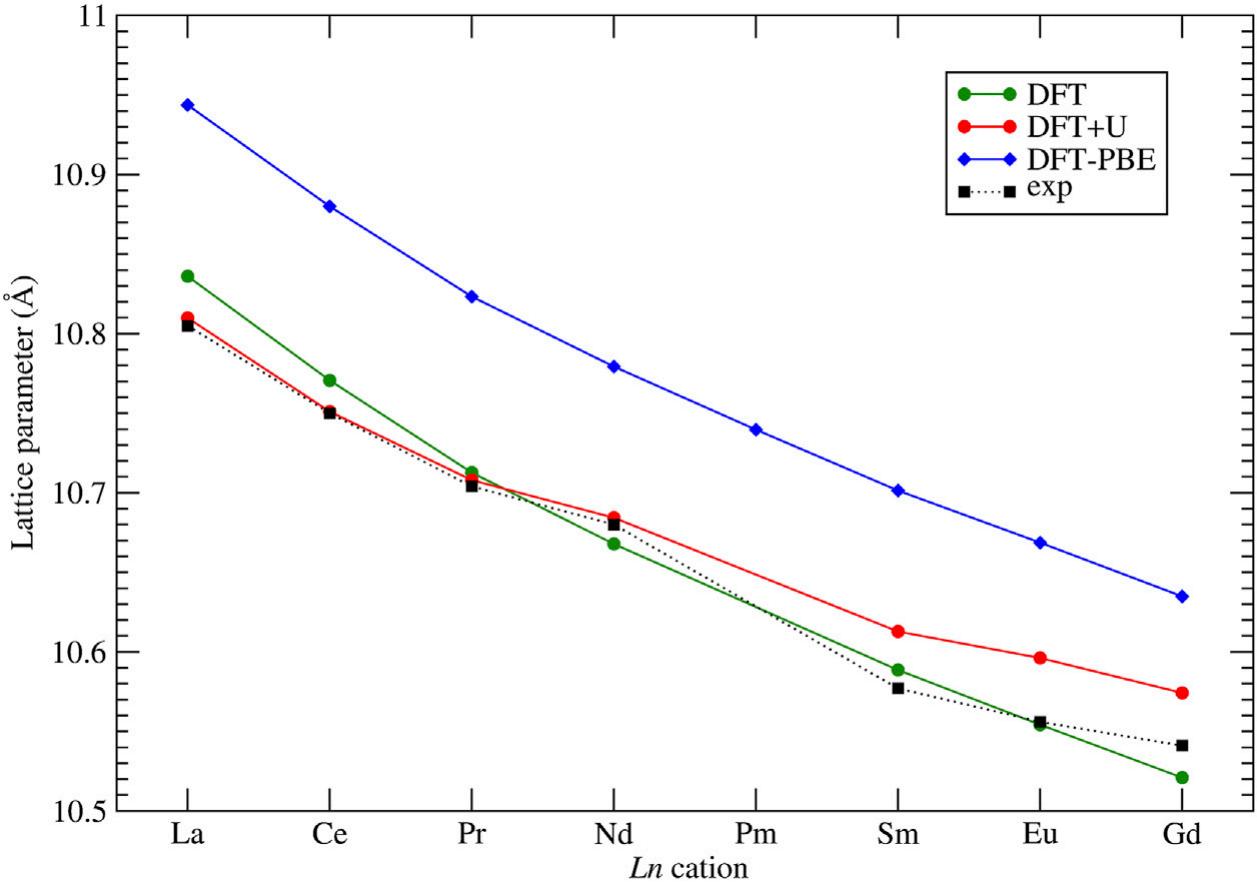
Computed with the DFT *”f in the core”* and DFT + *U* methods, and measured at ambient conditions lattice parameters of series of *Ln*_2_Zr_2_O_7_ pyrochlores. Experimental data come from Harvey et al. (2005); Koohpayeh et al. (2014); Vaisakhan Thampi et al. (2017); Hagiwara et al. (2013); Mandal et al. (2007); Nästren et al. (2009); Sasaki et al. (2004). The lines connecting the data points are plotted as eye guidance to show the trends.

An important test of a computational method is its ability to reproduce the measured lattice parameters of computed crystalline solids. Although some studies report excellent match of the computed lattice parameters of pyrochlores to the measured values (Feng et al., 2011), our previous studies have shown that the structural parameters of lanthanide-orthophosphates are very sensitive to the applied computational method, especially to the exchange-correlation functional (Blanca-Romero et al., 2014). A correct computation of strongly correlated 4*f* electrons also plays an important role in those cases. The lattice parameters of considered stoichiometric pyrochlores have been measured at ambient conditions by different studies (Sasaki et al., 2004; Harvey et al., 2005; Mandal et al., 2007; Nästren et al., 2009; Hagiwara et al., 2013; Koohpayeh et al., 2014; Vaisakhan Thampi et al., 2017). These are collected in Figure 2 and compared to the computed data. With our computational setup, applying the PBEsol exchange-correlations functional, we got excellent match to the measured lattice parameters of all the considered stoichiometric zirconate pyrochlores, with a difference within $∼0.04\;\overset{{}^{\circ}}{A}$ . We note that thermal expansion contributes to this difference at the level of $∼\;0.01\;\overset{{}^{\circ}}{A}$ (see Section 3.1.3). Such a good result is very important when, for instance, analyzing the structural change upon doping pyrochlores with actinide elements (Section 3.1.3). On the other hand, it is clear that the PBE exchange-correlation functional overestimates the lattice parameter by $∼\;0.12\;\overset{{}^{\circ}}{A}$ .

#### 3.1.2 Non-Stoichiometric Compositions: Nd_x_Zr_1-x_O_2−x/2_

The lattice parameter of non-stoichiometric fluorite and pyrochlore compounds can be predicted with an ion-close-packing approach by taking into account charges and coordination numbers of *A* and *B* cations and the presence of oxygen vacancies (Ohmichi et al., 1981; Hong and Virkar, 1995; Marrocchelli et al., 2012, 2013; Bukaemskiy et al., 2021). The fluorite lattice parameter, *a*, can be expressed as a function of the average radii of cations and anions as follows

$$a=\frac{4}{\sqrt{3}}\left(R_{c}+R_{a}\right),$$ (1)

where *R*_*c*_ and *R*_*a*_ are additive sums of contributions from all types of cations and anions. The pyrochlore lattice parameter is twice the fluorite lattice parameter. It has been recognized that a vacancy can be viewed as an anion species with a certain effective radius. Models, which apply this approach to fluorite compounds are divided into two groups. In the first group, the vacancy radius is found to be significantly smaller than the radius of an oxygen anion (Hong and Virkar, 1995; Marrocchelli et al., 2012; Marrocchelli et al., 2013), in the second group the vacancy is significantly larger than the oxygen anion (Ohmichi et al., 1981; Bukaemskiy et al., 2021). As discussed recently by Bukaemskiy et al. (2021), the distinction is due to taking or not taking into an account a change in the average cation coordination due to the insertion of vacancies. If the average cation coordination is counted properly as 8 − 2*x*, then the vacancy radius is predicted to be about 11% larger than the ionic radius of O^2−^. The later approach is shown to be consistent with the results of *ab initio* simulations (Stapper et al., 1999; Bukaemskiy et al., 2021). The loss of the negative charge of an oxygen anion (due to a vacancy formation) pushes the four cations that form tetrahedron encapsulating the 8*a* vacancy (Figure 1) away by ∼ 0.18 Å, indicating the vacancy size to be larger than O^2−^. On the other hand, the six nearest oxygen atoms move towards the vacancy (and to the four cations nearest to the vacancy) by $∼0.24\;\overset{{}^{\circ}}{A}$ . In the first group of models, both the extension and the contraction effects are mapped onto the vacancy size thus making its radius smaller than that of O^2−^. In the second group of models, the latter contraction effect is taken into account via a change in the cation radii. The coordination numbers of the four cations are decreased due to the vacancy formation, consequently their cation radii decrease. The average cation coordination number in the stoichiometric pyrochlore is thus 7 (8 – 2*x* for *x* = 0.5), while it is eight in the ideal fluorite (*x* = 0). Models that take into account the change in cation coordination allow a more accurate description of the lattice parameter variation in fluorite and pyrochlore compounds, because they are sensitive not only to changes in the average composition, but also to short- and long-range order effects. For example, due to the long-range order in pyrochlore, the coordination numbers of *A* and *B* cations appear to be significantly different from the average value of 7. The *B* cation is 6-fold coordinated, while the *A* cation is 8-fold coordinated. As shown by Bukaemskiy et al. (2021), the tendency of *B* cations to lower their coordination number, i.e. to attract vacancies, can be deduced to exist at non-stoichiometric compositions as well. For example, the lattice parameter variation in *A*_*x*_ *B*_1−*x*_O_2−0.5*x*_ systems (*A* = *Ln*, Y; *B* = Zr) within the interval of 0 < *x* < 1/3 can be explained under the assumption that all vacancies are fully surrounded by *B* cations and all *A* cations keep their coordination equal to 8. It is further shown that this strategy breaks down at *x* > 1/3, when the amount of Zr is insufficient for building up the surrounding of isolated vacancies. Within the interval of 1/3 < *x* < 0.5 two different models of SRO develop, one of which is consistent with the type of order in pyrochlore. The tendency of a (large) vacancy to reside close to a *B* cation is valid only in systems in which a *B* cation is significantly smaller than an *A* cation. When the radii of *A* and *B* cations are approximately equal, the electrostatic tendency of a vacancy association to an *A* (III) cation becomes more important. This is confirmed by the *ab initio* simulations (Bogicevic et al., 2001; Bogicevic and Wolverton, 2003; Solomon et al., 2016).

Finkeldei et al. (2017) investigated the series of Nd_x_Zr_1-x_O_2-x/2_ compositions by applying XRD and calorimetric measurements, and *ab initio* modeling methods. The XRD data show gradual, linear decrease in the lattice parameter upon lowering of Nd content with *x*, showing slightly different slopes within fluorite (0.23 < *x* < 0.30) and pyrochlore (0.33 < *x* < 0.53) domains. The observed trend for pyrochlore compounds has been well reproduced by the computed data, applying a structural model in which with gradual decreasing of Nd content, the oxygen vacancies are randomly filled with oxygen atoms.

#### 3.1.3 Nd_2_Zr_2_O_7_ Pyrochlore Doped With Actinides

When stoichiometric pyrochlore is doped with actinide elements, the main questions are the incorporation site (*A* or *B* cation) and the oxidation state of the dopant. The experimental data on Nd_2_Zr_2_O_7_ pyrochlore of Nästren et al. (2009) and Finkeldei et al. (2020) indicate doping at the *A* cation site (Nd), even when the oxidation state of *An* cation is larger than 3+ . In order to shed light on this phenomenon we performed calculations of the lattice parameter of a series of Nd_2_Zr_2_O_7_ pyrochlores doped with different actinide elements (*An* = U, Np, Pu, Am, Cm). In the first step we computed the Hubbard *U* parameters for actinides incorporated into the Nd-pyrochlore at different cation sites and in different oxidation states. The values are listed in Table 2. The computed values are consistent with the results of our previous studies (e.g., Beridze and Kowalski (2014); Beridze et al. (2016); Kvashnina et al. (2018); Sun et al. (2021)). There is an increase in the Hubbard *U* parameter with the oxidation state of *An* cation, and a general increase along actinide series, which is similar to the trend obtained for the *Ln* series (Table 1). This has an impact on the lattice parameters of *An*-doped pyrochlores.

**TABLE 2:** The computed Hubbard *U* parameters for *An* dopants, in different oxidation state on Nd site and as tetravalent species on Zr site, in Nd_2_Zr_2_O_7_ pyrochlore. Values are in eV.

Actinide	*An* (III)	*An* (IV)	*An* (IV) on Zr	*An*(V)
U	-	1.7	2.3	2.2
Np	-	2.1	2.0	2.6
Pu	2.4	2.6	2.6	
Am	2.6	3.3	3.1	-
Cm	1.7	3.8	3.3	-

**FIGURE 3:**
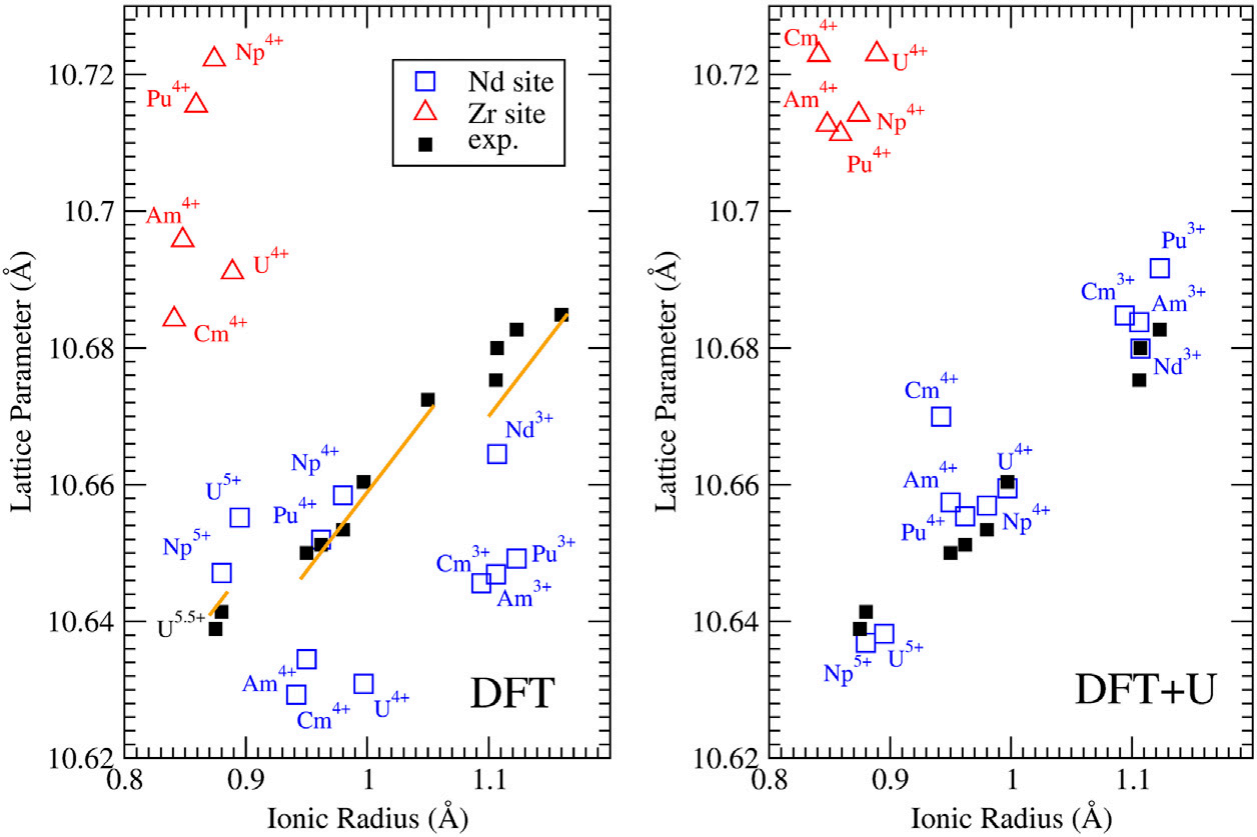
Computed with the DFT *”f in the core”* (left panel) and DFT + *U* (right panel) methods, and measured lattice parameters for Nd_2_Zr_2_O_7_ doped with various actinides on Nd (blue squares) and Zr (red triangles) sites. The experimental data collected at ambient conditions (black filled squares) are those of Nästren et al. (2009). The orange solid lines represent prediction of ion-close-packing model of Bukaemskiy et al. (2021). The model predicts three different linear dependencies that correspond to the cases of +3, +4 and +5 *An* cations substituting on the Nd site.

Figure 3 shows the computed and measured (Nästren et al., 2009) lattice parameters of Nd_2_Zr_2_O_7_ pyrochlore doped with different actinides. In order to be consistent with the measurements of Nästren et al. (2009), all the computed values were re-scaled to actinide stoichiometries of 0.2 per *A*_2_ *B*_2_O_7_ formula unit and were corrected for thermal expansion using the linear thermal expansion coefficient (TEC) and assuming ambient temperature of 298 K. There is some disagreement in the literature about the low temperature (from 0 K up to 300 K) TEC of neodymium zirconate pyrochlores (Kutty et al., 1994; Shimamura et al., 2007; Liu et al., 2010; Feng et al., 2012; Guo et al., 2015) (range from 5 ⋅ 10^–6^ K^−1^ to 12 ⋅ 10^–6^ K^−1^). Here we applied the representative value of 9.11 10^–5^ K^−1^ determined by Kutty et al. (1994). We note, however, that due to the uncertainty in the TEC, the lattice parameters values computed here could have an additional error of ∼ 0.01 Å. Nevertheless, with the selected TEC we perfectly reproduce the measured lattice parameter of stoichiometric Nd_2_Zr_2_O_7_ pyrochlore, which is essential when predicting the change in the lattice parameter upon incorporation of actinide elements.

The measurements of Nästren et al. (2009) show a clear linear-like relationship between the lattice parameter of *An*-doped Nd_2_Zr_2_O_7_ and the ionic radius of the *An* cation. Figure 3 shows that when the *f* electrons are not computed explicitly, this trend is captured only on the qualitative level. In contrast, the lattice parameters computed with the parameter-free DFT + *U* method used here, with the Hubbard correction applied to *An* and *Ln* elements, successfully reproduce the linear relationship identified by Nästren et al. (2009). This shows the importance of electronic correlations on reliable and accurate computation of lattice parameters of *f* elements containing oxides. The calculations show that tetravalent and pentavalent actinides, when doped on the Nd^3+^ sites, cause a significant lattice contraction, while tetravalent actinides when doped on the Zr^4+^ site lead to significant lattice expansion. The later effect occurs because 6-fold coordinated Zr^4+^ has the ionic radius of only 0.72 Å (Shannon, 1976), which is significantly smaller than that of the considered *An* cations. As the measurements of Nästren et al. (2009) show a lattice contraction for all tetra and pentavalent actinides, the excellent match between the values computed here and the experimental data supports their interpretation that the actinides cations are incorporated on the Nd-site.

On the computational side, the DFT + *U* method improves accuracy of the predicted lattice parameters, reducing the average error from 0.017 Å (obtained with the simple DFT-PBEsol approach (*f in the core*) to just 0.005 Å (i.e. less than 0.1*%*), which is an unprecedented accuracy having in mind that 4*f* and 5*f* elements are computationally challenging. Pu^3+^ is also correctly predicted to expand the lattice. Furthermore, the prediction of the lattice parameters of pristine material (Nd^3+^) and that of Pu^3+^ doped systems have relative errors of only 0.001 and 0.09*%*, respectively. Even considering the uncertainty in the TEC, these are excellent results showing that the parameter free DFT + *U* method is a highly reliable tool for predicting lattice parameters in the actinide-pyrochlore system.

An interesting observation is that doping with Cm^4+^ produces a significantly greater lattice parameter than with Am^4+^, breaking the linear relationship followed by the other *An* cations’ cases. This is not expected as Cm^4+^ has smaller ionic radius than Am^4+^. We note, however, that such a subtle differences have been observed due to so-called tetrad effect associated with the number of electrons in *f* shells (e.g., Wilden et al. (2019)). On the computational side, the reason for this is a significantly larger Hubbard *U* parameter value derived for Cm^4+^ than that for Am^4+^ or Cm^3+^ (Table 2). This resembles the case of Eu^3+^ and Gd^3+^ (Table 1) and results from the strongest electronic correlation effects for cations with nearly complete *f* half-shell (i.e., with 6 *f* electrons). Unfortunately, we do not have experimental data for Cm doped Nd_2_Zr_2_O_7_ in the data set of Nästren et al. (2009) to confirm our claim. Confirmation of such effect would be a direct demonstration of impact of electronic correlation effects on the structural arrangements in *f* elements oxides.

Empirical models can be applied to predict the change in lattice parameters of doped materials. The ion-close-packing model of Bukaemskiy et al. (2021) provides information on the structural effects occurring in a doped compound purely due to charges in coordination numbers of the constituent cations. Figure 3 shows the variation of lattice parameters of actinide-bearing Nd-pyrochlore computed with the ion-packing model, utilizing the Shannon’s cation radii (Shannon, 1976). The model provides values that are in good agreement with the experimental lattice parameter data and the *ab initio* calculations discussed above (although it can not capture the discussed, subtle effects resulting from the electronic correlations). This is yet another confirmation of the *A* cation site doping scenario irrespectively of the valence state of *An* cations. On the other hand, the empirical model suggests that the linear relationship observed by Nästren et al. (2009) between the lattice parameter and the radius of *An* cation is split into three linear trends corresponding to +3, +4 and +5 cations.

### 3.2 Formation Enthalpies

#### 3.2.1 Stoichiometric Pyrochlore: Order-Disorder Transition

The formation enthalpies from oxides for series of stoichiometric pyrochlore-type compounds were measured by Helean et al. (2004) (Ln_2_Ti_2_O_7_), Lian et al. (2006a) (Ln_2_Sn_2_O_7_), Ushakov et al. (2007) (Ln_2_Hf_2_O_7_) and Radha et al. (2009); Saradhi et al. (2012) (Ln_2_Zr_2_O_7_). Kowalski (2020) derived these values with the DFT-based atomistic modeling methods. He was able to reproduce the experimentally seen trends in the behavior of formation enthalpies along the lanthanide series with the derived values within 20 kJ/mol. Accurate computation of formation enthalpies is crucial for predicting other important parameters, such as for instance, the order-disorder transition temperature (Jiang et al., 2009; Li et al., 2015) and the extend of ordering in disordered pyrochlore compounds.

Atomistic modeling studies have been applied to predict the order-disorder transition temperature (*T*_*O*−*D*_) of series of stoichiometric pyrochlore compounds. This parameters is usually computed assuming simple relationship between the enthalpy Δ*H* and entropy Δ*S* of disordering process:

$$T_{O-D}=\frac{\Delta H}{\Delta S},$$ (2)

and an estimate of the disordering entropy assuming a complete disordering of the disordered phase (e.g., Jiang et al. (2009)). Earlier atomistic modeling studies derived this parameter by applying simple force-fields to describe interatomic interactions (Minervini et al., 2000; Sickafus et al., 2000). Jiang et al. (2009) applied DFT to derive *T*_*O*−*D*_ and obtained values that are smaller by ∼300 K from the measured values, but the experimentally observed trend in decrease of transition temperature along lanthanide series has been well captured. The main reason for this offset is the application of the standard DFT method with the *f* electrons included into the pseudopotential core and not computed explicitly. Li et al. (2015) have shown that the prediction of *T*_*O*−*D*_ is significantly improved when *f* electrons are computed explicitly and the electronic correlations computed with the DFT + *U* method with the Hubbard *U* parameter derived from first principles using the linear response method (Cococcioni and de Gironcoli, 2005). They got a nearly perfect agreement with the measured *T*_*O*−*D*_ for Sm_2_Zr_2_O_7_, Gd_2_Zr_2_O_7_, Gd_2_Hf_2_O_7_ and Tb_2_Hf_2_O_7_ compounds.

The aforementioned computed results may be seen surprising in view of the more recent finding of significant SRO in this class of compounds (Shamblin et al., 2016). We notice however, that a SRO is accompanied by a simultaneous decrease in the disordering enthalpy and entropy, as demonstrated by Kowalski (2020). This results in preservation of constancy of the ratio given in Eq. 2. Furthermore, as shown by Finkeldei et al. (2017) for Nd_2_Zr_2_O_7_, the correct prediction of *T*_*O*−*D*_ may require application of more complex model of disordered phase. In that study a simple two-state statistical model has been applied. Results of applying such a model to selected pyrochlore compounds is given in Table 3. We note that the prediction could be improved with: 1) a better computational setup (explicit computation of *f* electrons, Li et al. (2015)) and 2) with a better structural model of disordered pyrochlore, and disordering entropy in such a system. The later would require more advanced thermodynamic modeling approach.

**TABLE 3:** The *T*_*O*−*D*_ in K computed with different methods: modeling disordered phase by defect fluorite (PY-DF), weberite (PY-WB) and defect fluorite/weberite mixture by the two state model of Finkeldei et al. (2017) (PY-WB/DF), and measured for selected pyrochlore compounds. In parentheses we provide the estimated configurational entropy of disordered phase in J/K/mol.

Compound	Nd_2_Zr_2_O_7_	Gd_2_Hf_2_O_7_
PY-DF	2,310 (48.1)	2,252 (48.1)
PY-WB	3,452 (11.5)	2039 (11.5)
PY-WB/DF	2,405 (38.1)	2,327 (26.3)
exp	2,573	2,723

The extend of SRO in stoichiometric defect fluorite compositions has been demonstrated experimentally with calorimetric measurements (Ushakov et al., 2007; Saradhi et al., 2012; Finkeldei et al., 2017) and neutron scattering data (Shamblin et al., 2016). The later data are best fitted with a weberite-type structural representation, in which SRO is emulated via a long-range ordered phase that includes both ordered and disordered sublattices. This finding has been supported by atomistic simulations of Kowalski (2020) who has shown that formation enthalpies computed with the weberite-type structural model are in good agreement with the aforementioned calorimetric measurements and that fully disordered defect fluorite structure is thermodynamically unfeasible. The fact that weberite-type structural model fits nicely the data, however, is not a direct proof that this model gives the best possible characterization of cation and anion distribution in the disordered pyrochlore. Theoretical considerations lead to other possible structural arrangements (Bukaemskiy et al., 2021; Vinograd and Bukaemskiy, 2021). The weberite model may not be the best solution for the configurational entropy of the disordered phase, as the nominal entropy effect of a pyrochlore/weberite transformation (11.52 J/K/mol) seems too small to fit the calorimetric data for Eu_2_Zr_2_O_7_ (18 J/K/mol, Saradhi et al. (2012); Finkeldei et al. (2017)). Nevertheless, the data for Gd_2_Hf_2_O_7_ (12 J/K/mol, Ushakov et al. (2007)) seem to be consistent with the weberite scenario. The recent simulation study of Vinograd and Bukaemskiy (2021) provided a thermodynamic model for the disordered fluorite, which counts the configurational entropy contributions only from *A* and *B* cations occurring in the same coordination. The model assumes that the disordered fluorite is characterized by an ordered distribution of vacancies and a random distribution of *A* and *B* cations that all occur in the 7-fold coordination. The resulting nominal entropy of 23.04 J/K/mol is larger than the aforementioned experimental data, but such a model can not be easily rejected considering the likely presence of a degree of disorder in measured pyrochlore samples, which would lead to underestimation of disordering entropy by the calorimetry studies. Nevertheless, the model of Vinograd and Bukaemskiy (2021) provides a semi-quantitative description of the solution calorimetry data at non-stoichiometric compositions of *A*_*x*_ *B*_1−*x*_O_2−0.5*x*_ systems with *A* = Sm, Gd, Dy and Y. It predicted the stabilization of various types of short and long-range order, depending on the temperature and the composition.

#### 3.2.2 Non-Stoichiometric Pyrochlore: Nd_x_Zr_1-x_O_2−x/2_

Finkeldei et al. (2017) studied the composition-induced disordering in Nd_x_Zr_1-x_O_2-x/2_ pyrochlore by applying XRD, calorimetric and atomistic modeling methods. On experimental side they were able to detect the formation of a disordered fluorite phase at *x* = 0.31 (corresponding to Zr/Nd ratio of 2). This has been clearly indicated by a small change in the lattice parameter vs. *x* slope, within a narrow transition region in which the two phases with slightly different lattice parameters coexist, and by a significant increase in the measured formation enthalpy of ∼30 kJ/mol, which corresponds to the disordering entropy of ∼16 J/K/mol. The later value is significantly smaller than the value expected for the fully disordered defect fluorite phase of ∼24 J/K/mol and clearly indicates the presence of a significant SRO. Finkeldei et al. (2017) introduced a disordering model in which, upon decrease of Nd content, vacant sites are gradually and randomly occupied by oxygen atoms (coming from excess of ZrO_2_). They were able to reproduce well the change in the lattice parameter and formation enthalpy of pyrochlore phase, but significantly overestimated the formation enthalpy of defect fluorite compounds series. Following experimental finding of weberite-type SRO by Shamblin et al. (2016), Finkeldei et al. (2017) modelled the disordered phase as a statistical mixture of weberite and ideally disordered defect fluorite. With this approach, the measured data could be reproduced reasonably well, which shows yet another evidence for significant SRO in this class of compounds and demonstrates that weberite is a reasonable model for this phenomenon. On the other hand, these simulations have shown that weberite model, providing a good structural description of local ordering in defect fluorite, may not be a perfect model for predicting configurational entropies, implying the need for an additional (more disordered) structural component.

### 3.3 Defect Formation Energies

**FIGURE 4:**
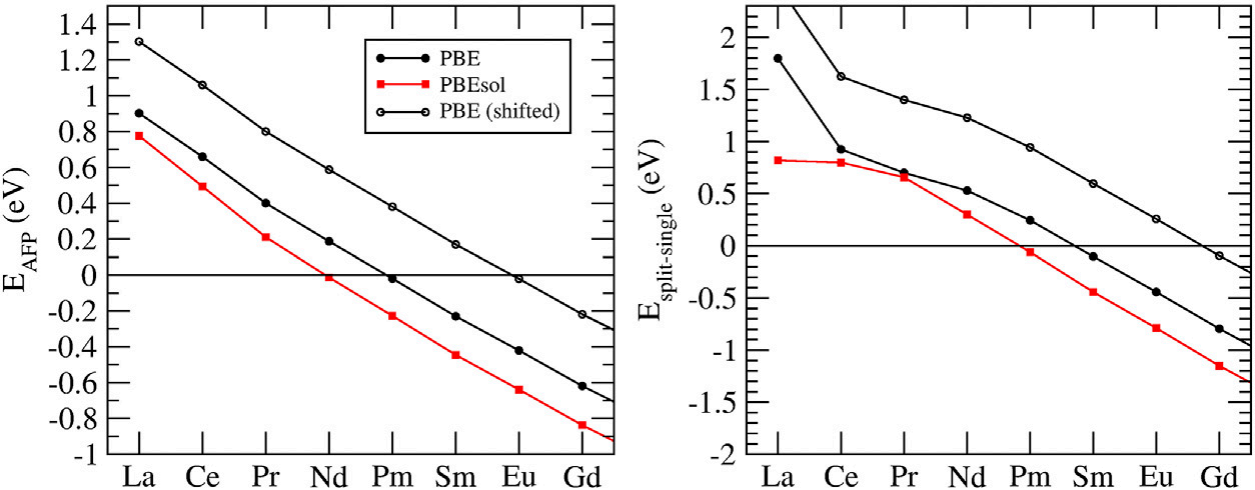
Anion Frenkel pair defect formation energy (*E*_*AFP*_, left panel) and the difference in the energy between split and single vacancy configurations (*E*_*split*−*single*_, right panel) in a series of *Ln*_2_Zr_2_O_7_ pyrochlores. The values were computed with the PBE (black filled circles) and PBEsol (red filled squares) exchange-correlation functionals. The black open circles show the predictions with *E*_*AFP*_ adjusted by 0.4 eV to match the measurements of Maram et al. (2018), as discussed in Section 3.4.

Besides formation enthalpies, several atomistic modeling studies focused on understanding the formation of defects in pyrochlore compounds (Minervini et al., 2000; Sickafus et al., 2000; Li et al., 2015; Li and Kowalski, 2018). The ability of a material to accumulate defects is considered as a descriptor of radiation damage resistance (Sickafus et al., 2000; Sickafus et al., 2007). Two main defects have been considered, cation-antisite (flipping positions of cations *A* and *B*) and anion Frenkel pair (moving an 48*f* oxygen to an 8*a* vacant site). In order to understand the tendency of a material to accumulate defects, Sickafus et al. (2000) computed cation antisite defect formation energies using force-field-based description of interactions. They showed that compounds that are prone to transition to defect fluorite have cations *A* and *B* of similar sizes, and resulting lower cation antisite defect formation energy. In more accurate DFT-based simulations, Li et al. (2015) have found a good correlation between the anion-Frenkel pair defect formation energy and formation of defect fluorite. For the compounds that form disordered phase, or transfer to it at temperature, the anion Frenkel pair defect formation energy is small or negative, which is indicated in Figure 4. The follow up experimental studies of Maram et al. (2018) confirmed this prediction. Li and Kowalski (2018) demonstrated that a combination of cation antisite and anion Frenkel pair defects results in lowering of the resulting defect formation energy and barrier for oxygen migration. This strongly indicates that mobility on the oxygen sublattice is responsible for the disordering and enhanced radiation damage resistance of selected pyrochlores (e.g., Gd_2_Zr_2_O_7_).

### 3.4 Ionic Conductivity

Zirconia-based materials, including pyrochlore, are considered as prospective ionic conductors for solid state electrolytes (Wuensch, 2000; Diazguillen et al., 2008; Kumar et al., 2008; Mandal et al., 2008; Shlyakhtina and Shcherbakova, 2012; Anithakumari et al., 2016). One of the striking examples is yttria-stabilized zirconia - one of the fastest solid state ionic conductors known (Kowalski et al., 2021). The oxygen conduction in a stoichiometric pyrochlore occurs due to jumps of oxygens, or oxygen vacancies, between the neighboring 48*f* sites. In stoichiometric pyrochlore these sites are fully occupied. In order thus to allow for oxygen diffusion, a vacancy must be formed on a 48*f* site. This occurs via transfer of an oxygen atom to a 8*a* vacant site (Pirzada et al., 2001; Li and Kowalski, 2018). When such a vacancy configuration forms, it can migrate through the 48*f* lattice. van Dijk et al. (1985) realized that in addition to a simple vacancy on 48*f* site, a split vacancy state could be more stable for pyrochlores with heavier lanthanides. In such a state, a 48*f* oxygen ion adjacent to the 48*f* vacancy moves to an interstitial location displaced symmetrically between the two vacant 48*f* sites and the adjacent 8*a* site (Pirzada et al., 2001). The energy difference between the two vacancy configurations computed here is illustrated in the right panel of Figure 4. The results indicate stabilization of split vacancy state for Sm and heavier lanthanides. However, the result depends significantly on the computational setup, for instance on the exchange-correlation functional. The difference between energies computed with the PBE and PBEsol functionals is ∼ 0.4 eV. We note that there is a correlation between the energy of formation of anion Frenkel pair and the energy difference between the two vacancy configurations, Δ*E*_*split*−*single*_ ∼ 1.8*E*_*AFP*_ + 0.3 eV. Maram et al. (2018) measured the energy of formation of anion Frenkel pair defect of ∼0.1–0.3 eV for Sm_2_Zr_2_ *O*_7_, Eu_2_Zr_2_O_7_ and Gd_2_Zr_2_O_7_. These data, although measured indirectly and at high temperatures, and thus also highly uncertain, would indicate that the computed values shown in left panel of Figure 4 are underestimated by as much as ∼0.4−0.9eV . If so, the computed energy difference between the two vacancy states could be also underestimated by as much as ∼ 0.7–1.6 eV, which would indicate formation of split vacancy state for lanthanides heavier than Sm, e.g. after Eu, as illustrated in Figure 4.

Simulations of Li and Kowalski (2018) show good match of the computed activation energy to the values measured by various studies. Li and Kowalski (2018) and present studies have shown that the split vacancy state forms for zirconate pyrochlores with heavier lanthanides. This splitting state substantially increases the activation barrier for ionic conductivity (Li and Kowalski, 2018). Series of calculations of zirconate pyrochlores with different *Ln* cations performed here show that the barrier for oxygen migrations is ∼ 0.7 eV and ∼ 1.2 eV for single and split vacancy configurations, respectively (Table 4). The computed activation barriers, taken as a sum of the barrier for oxygen diffusion and the anion Frenkel pair defect formation energy (Li and Kowalski, 2018), are reported in Table 4. The decrease in the ionic conductivity and the related increase in the activation energy in Gd_2_Zr_2_O_7_ (by ∼0.3eV with respect to Eu_2_Zr_2_O_7_) as reported by Yamamura (2003) is thus a direct manifestation of the split-vacancy state formation. The difference in the computed activation energy between Gd_2_Zr_2_O_7_ and Eu_2_Zr_2_O_7_ of ∼0.3eV is well consistent with the experimental results.

**TABLE 4:** The activation energy (simulated, *E*_*a*,*sim*_; measured *E*_*a*, exp_, Yamamura (2003)) and energy barriers (*E*_*b*_) for oxygen migrations in series of *Ln*_2_Zr_2_O_7_ pyrochlores. The energy barriers for 48*f*-48*f* diffusion path for single (*E*_*b*,*single*_) and split (*E*_*b*,*split*_) vacancy states, as well as barrier for oxygen migration from 48*f* to 8*a* site (*E*_*b*,8*a*_) are reported. The second set of experimental values represent the results of our fits to the data of Yamamura (2003). Values are in eV.

Compound	*E*_*a*,*sim*_	*E*_*a*, exp_	*E*_*b*,*single*_	*E*_*b*,*split*_	*E*_*b*,8*a*_
La	-	0.56/0.63	0.74	-	-
Ce	1.39	-	0.74	-	0.69
Pr	1.13	-	0.73	-	0.49
Nd	0.91	0.68/0.61	0.73	-	0.38
Pm	0.69	-	0.71	1.17	0.31
Sm	0.46	0.56/0.71	0.69	1.21	0.24
Eu	0.28	0.58/0.61	-	1.23	0.19
Gd	0.58	0.88/0.94	-	1.21	0.14

### 3.5 Radiation Damage

Radiation damage resistance of pyrochlore compound is an important property for application of these materials as immobilization matrices for radionuclides. For pyrochlores, this property is associated with the tendency of a compound to disorder. The pyrochlore compounds like Gd_2_Zr_2_O_7_ show enhanced resistance to radiation, forming disordered solid phase (defect fluorite) under irradiation, while other compounds, such as Gd_2_Ti_2_O_7_ amorphize (Ewing et al., 2004). There have been a few atomistic modeling studies aiming at understanding of this disordering-induced radiation damage resistance process (e.g., Crocombette and Chartier (2007); Chartier et al. (2003); Crocombette et al. (2006); Chartier et al. (2009); Chartier et al. (2002)). These aimed at delivering parameters such as critical amorphization dose (irradiation dose at which amorphous phase is reached), critical temperature (above which irradiation-induced amorphization does not happen due to self-healing behavior), and at understanding of structural changes upon irradiation.

**FIGURE 5:**
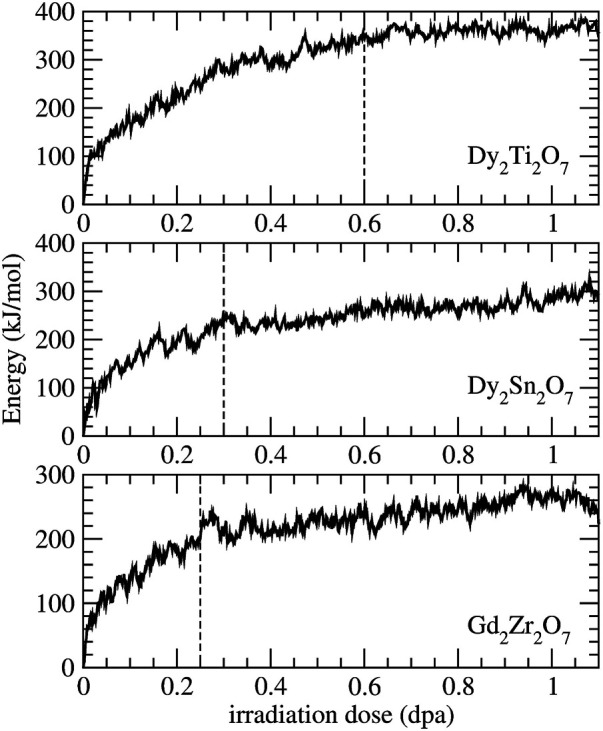
Simulated radiation damage effect on the internal energy for Dy_2_Ti_2_O_7_, Dy_2_Sn_2_O_7_ and Gd_2_Zr_2_O_7_ pyrochlores. The vertical dashed lines indicate the simulated critical amorphization doses.

Recently, using classical molecular dynamics simulations Sun et al. (2021) successfully simulated radiation damage effects in a series of borosilicate glasses. With a defect accumulation procedure they were able to deliver information on critical irradiation dose, Young’s modulus and stored internal energy that are consistent with the measured data. Here we applied a similar procedure to derive the critical amorphization dose and amorphization energy of the Gd_2_Zr_2_O_7_, Dy_2_Sn_2_O_7_ and Dy_2_Ti_2_O_7_ pyrochlores, the last two measured by Chung et al. (2018a,b). The results are given in Figure 5 and Table 5. The reported change in energy with irradiation dose is consistent with previous simulations of La_2_Zr_2_O_7_ (Crocombette et al., 2006), with similar values for the amorphization energy ( ∼ 170–240 kJ/mol, Crocombette et al. (2006)) and critical irradiation dose (∼ 0.25 dpa, Crocombette et al. (2006)). We were able to reproduce well the measured critical amorphization dose of ∼0.3 dpa in the case of Dy_2_Sn_2_O_7_ (Ewing et al., 2004). The computed amorphization energy for this compound is also consistent with the measured value of 284 ± 6 kJ/mol (Chung et al., 2018a). On the other hand, in the case of Dy_2_Ti_2_O_7_, the simulations overestimate the critical dose ( ∼0.6 dpa vs. ∼0.25 dpa, Lian et al. (2003)) and the amorphization energy. For Gd_2_Zr_2_O_7_ we also obtained amorphization at ∼0.25 dpa, while this compound in reality transfers to disordered crystalline phase. The reason for such a discrepancy is the applied simplified description of the interatomic interactions in the performed simulations. The radiation damage tendencies in pyrochlores are correlated to the energy cost of defects formation in their crystalline lattice. In Table 5 we list the anion Frenkel pair defect formation energy as predicted with the applied here empirical force field parameterization of Minervini et al. (2000); Stanek et al. (2002). These energies are significantly larger than these predicted with the *ab initio* calculations (Li et al., 2015) or measurements of Maram et al. (2018). It is thus not surprising that with the applied force fields we are able to amorphize all the considered compounds. On the other hand, the trend in the anion Frenkel pair defect formation energies reflects the order of measured amorphization energies. The negative or small value for Gd_2_Zr_2_O_7_ would also indicate transition to disordered phase and high resistance to radiation-induced amorphization. The importance of applying correct force fields in simulations of radiation damage is discussed in Sun et al. (2021). Our results strongly suggest that for reliable simulations of radiation damage effects in pyrochlores, the applied force-fields must be reparameterized. We also note that it is difficult to directly compare the simulated and experimental data on irradiated pyrochlores. This is because of significantly different irradiation doses in simulations, experiments and these expected under nuclear waste storage conditions, as well as due to simplistic, ballistic description of the radiation damage by the applied atomistic simulations (Chartier et al., 2009; Sun et al., 2021).

**TABLE 5:** The simulated (*H*_*sim*_) and measured (*H*_exp_, Chung et al. (2018a) and Chung et al. (2018b)) amorphization enthalpy and critical amorphization dose (*AD*) (Ewing et al., 2004) in selected pyrochlores. The values of enthalpies are in kJ/mol. The Anion Frenkel Pair defect formation energy computed with the force fields (*E*_*AFP*,1_, Minervini et al. (2000)) and DFT simulations (*E*_*AFP*,2_, Li et al. (2015)) are given in eV.

Compound	*H*_*sim*_	*H*_exp_	*AD*_*sim*_ (dpa)	*AD*_exp_ (dpa)	*E*_*AFP*,1_	*E*_*AFP*,2_
Dy_2_Ti_2_O_7_	364	243 ± 6	0.6	0.25	10.0	1.0
Dy_2_Sn_2_O_7_	282	284 ± 6	0.3	-	6.4	1.5
Gd_2_Zr_2_O_7_	257	-	0.25	-	4.8	-0.6

## 4 Conclusions

In this contribution we have presented an overview of our decade-long atomistic modeling research on pyrochlore-type ceramics. We discussed the molecular-level, atomistic simulations-based investigation of structural, thermodynamic, diffusion and radiation-induced properties that are of importance for applications of these materials in energy storage devices or as matrices for immobilization of actinides. We elucidate the importance of correct computation of strongly correlated *f* electrons. This is demonstrated by very accurate prediction of the lattice parameters of series of zirconate-pyrochlores doped with actinides, achieved with the parameter-free DFT + *U* method in which the Hubbard *U* parameter is derived from first principles. The comparison of the computed lattice parameter of Nd_2_Zr_2_O_7_ pyrochlore doped with different actinide elements to the measured data allows for a confident conclusion regarding the *An* doping site. Such an analysis clearly indicates that actinides are incorporated on Nd site, independently of their oxidation state (+3, +4 or +5). We discussed the role of atomistic modeling in understanding the formation of ordering in disordered pyrochlore phases. By computing the defect formation energies and migration barriers for oxygen atoms, we delivered insights into ionic diffusion in pyrochlore compounds. We explain the drop in the measured ionic conductivity for lanthanides heavier than Eu by the formation of the split vacancy states, which significantly increase the activation barrier for oxygen diffusion. Last but not least, with the classical molecular dynamics simulations, we derived the amorphization energy of selected, irradiated pyrochlore compounds and obtained a good qualitative match to the measured values of disordering enthalpies. The predicted critical amorphization doses are also reasonably well consistent with experiment. However, we highlight some essential differences between the simulated and measured data that are related to inaccuracies in the applied computational methods, including DFT approximations that are based on different types of exchange-correlation functionals, and to simplistic description of interatomic interactions by Buckingham-type interaction potentials that have been intensively used in the research on pyrochlores, especially in the investigation of irradiation effects. In-depth analysis of the measured data with the simulated results allows, on the one hand, for revealing the atomic-scale mechanism governing the investigated phenomena and on the other hand understanding the limitations of the applied computational methods. This should allow for further improvement of the predictive power of the atomistic simulation methods.

## Data Availability

The raw data supporting the conclusions of this article will be made available by the authors, without undue reservation.

## Footnotes

1 In this contribution we call DFT methods an *ab initio* approach as the exchange-correlation functionals applied in our studies, PBE and PBEsol, were designed based on pure-theoretical considerations.
